# Cbx3a/HP1γ Deficiency Disrupts Meiotic Progression and Triggers Germ Cell Apoptosis in Nile Tilapia

**DOI:** 10.3390/biom16081120

**Published:** 2026-07-31

**Authors:** Hongqin Jian, Jiahong Wu, Ruijuan Feng, Liang Zhang, Li Zhou, Xingyong Liu

**Affiliations:** Key Laboratory of Freshwater Fish Reproduction and Development (Ministry of Education), Key Laboratory of Chongqing Municipality for Aquatic Economic Animal Resources Conservation and Germplasm Creation, School of Life Sciences, Southwest University, Chongqing 400715, China; j18580681091@email.swu.edu.cn (H.J.);

**Keywords:** HP1γ, germ cell, meiosis, apoptosis, Nile tilapia

## Abstract

Heterochromatin Protein 1γ, encoded by the *Cbx3* gene, is a crucial epigenetic regulator that plays an essential role in mammalian meiotic progression. However, the functional divergence and conservation of this protein in teleosts—organisms possessing duplicated Cbx3 paralogs due to whole-genome duplication—remain to be elucidated. Building on previous research, we focused on *cbx3a* in Nile tilapia (*Oreochromis niloticus*), a significant aquaculture species and an excellent model for teleost reproductive studies, emphasizing its role in spermatogenesis. Expression analysis revealed that Cbx3a is localized to primordial germ cells and is sustained in spermatogonia, spermatocytes, and spermatids during spermatogenesis. CRISPR/Cas9-mediated knockout of *cbx3a* demonstrated that Cbx3a deficiency induces germ cell apoptosis, meiotic arrest, and sperm defects, including shortened tails and impaired motility, resulting in profound defects in sperm quantity and quality, strongly implying compromised male fertility. Transcriptomic analysis further identified dysregulated molecular pathways, including cytokine signaling and neuroactive ligand–receptor interactions. This provides novel mechanistic insights into HP1γ-mediated epigenetic regulation of meiosis. Notably, *cbx3a* mutants exhibited phenotypic bifurcation: a subset showed meiotic defects accompanied by sporadic germ cell apoptosis, whereas others underwent full meiotic arrest with pervasive germ cell apoptosis in adult gonads. Collectively, these findings clarify the essential and conserved role of Cbx3a/HP1γ in Nile tilapia spermatogenesis, thereby advancing the field of vertebrate reproductive epigenetics and providing a valuable theoretical basis for potential applications in reproductive management, such as improving sperm quality.

## 1. Introduction

Spermatogenesis, a fundamental process in reproductive biology, is meticulously regulated through multi-layered mechanisms involving genetic factors, hormonal signals (both endogenous and exogenous), and epigenetic modifications [1,2,3,4,5]. Epigenetic regulation, particularly through the dynamics of DNA methylation and histone modifications, constitutes a fundamental regulatory axis in germ cell development [6,7,8,9,10]. Heterochromatin Protein 1 (HP1), a highly conserved non-histone chromatin-binding factor, facilitates heterochromatin formation, thereby maintaining genomic stability and regulating gene expression in vertebrates [11,12,13]. In mammals, the HP1 family comprises three isoforms—HP1α, HP1β, and HP1γ—encoded by the Cbx5, Cbx1, and Cbx3 genes, respectively. Despite their high sequence homology, these isoforms exhibit significant divergence in subcellular localization and biological functions [14,15,16]. Notably, HP1γ (Cbx3), which is extensively involved in gene silencing, DNA damage repair, and cell cycle regulation [17,18,19], has emerged as a focal point in reproductive epigenetics due to its indispensable roles in spermatogenesis.

In the mammalian reproductive system, HP1γ plays a crucial role in gametogenesis through various mechanisms. Studies indicate that Cbx3 is constitutively expressed at high levels in male germ cells, including gonocytes and spermatocytes in mammals [20]. The functional loss of Cbx3 results in reduced embryonic viability and spermatogenic defects in mice [21]. Mechanistically, HP1γ recruits histone methyltransferases, such as G9a, to pericentric heterochromatin (PCH) regions, facilitating H3K9me2/3 modifications that stabilize meiotic chromosome synapsis [21]. Furthermore, the HP1γ-Aurora kinase signaling axis regulates mitotic division in spermatogenic cells and early embryonic cleavage; its dysfunction may lead to male infertility or developmental arrest [22,23,24]. Notably, Cbx3 is essential for the proliferation of primordial germ cells [25], underscoring its early regulatory role in establishing the germ cell lineage.

In contrast to mammals, teleosts possess two paralogous genes encoding HP1γ, namely Cbx3a and Cbx3b, which originated from the third round of whole-genome duplication (3R WGD) [26]. Phylogenetic analyses indicate that Cbx3a exhibits a greater degree of evolutionary conservation compared to its mammalian counterpart, Cbx3 [26]. Its mRNA is abundantly expressed in the gonadal tissues of species such as tilapia and ricefield eels [26,27]. However, the cellular localization and regulatory roles of Cbx3a in teleost gametogenesis remain to be elucidated.

The Nile tilapia (*Oreochromis niloticus*), a commercially significant cichlid fish belonging to the order Perciformes, serves as an ideal model for teleost reproductive research due to its tractable genetic manipulation system [28]. Our previous work has demonstrated that *cbx3a* is specifically upregulated, while *cbx3b* exhibits only basal expression during tilapia spermatogenesis [26], suggesting a predominant role for Cbx3a in this process. This study further reveals that Cbx3a protein localizes to the nuclei of germ cells at all spermatogenic stages, including spermatogonia, spermatocytes, and spermatids. CRISPR/Cas9-mediated mutation of *cbx3a* resulted in exacerbated apoptosis in germ cells, severe depletion of spermatogenic cells, and even ‘empty nest’ phenotypes devoid of germ cells. These findings underscore the essential role of Cbx3a in the proliferation, survival, and differentiation of male germ cells in teleosts. Our research not only broadens the functional understanding of HP1γ in vertebrate gametogenesis but also provides a theoretical foundation for the production of high-quality gametes in aquaculture.

## 2. Materials and Methods

### 2.1. Animals

Nile tilapia (*Oreochromis niloticus*) were reared in recirculating aerated freshwater tanks maintained at approximately 26 °C under a natural photoperiod. Animal experiments were conducted in accordance with the regulations outlined in the Guide for Care and received approval from the Institutional Animal Care and Use Committee at Southwest University, China (No. IACUC-20181015-12, 15 October 2018).

### 2.2. Generation of Cbx3a Mutation Line by CRISPR/Cas9

The nucleotide sequence of the *cbx3a* gene (Gene ID: 100694248) in Nile tilapia was retrieved from the NCBI database. In this study, CRISPR/Cas9 gene editing technology was utilized to successfully generate *cbx3a* mutants, with the target site precisely located within the second exon of the gene’s coding region. Specifically, synthesized guide RNA (gRNA, 250 ng/μL) and Cas9 protein (1 μg/μL, Invitrogen, USA) were co-injected into one-cell-stage embryos using a microinjection system (WPI PV830, USA). The injected embryos were maintained in a constant-temperature incubation system at 28 ± 0.5 °C until 10 days post-fertilization (dpf), after which they were transferred to a recirculating aquaculture system for standardized rearing. For mutant screening, F0 generation individuals were initially screened using a heteroduplex mobility assay (HMA) combined with Sanger sequencing. Positive F0 males were subsequently test-crossed with wild-type (WT) females to obtain F1 heterozygous mutants (*cbx3a*^+/−^). Through incrossing of the F1 generation, homozygous mutants (*cbx3a*^−/−^) were ultimately obtained. Consistent with Mendelian inheritance, which predicts a theoretical ratio of 25% for *cbx3a*^−/−^ mutants, the homozygous mutants established in this study carry a −2bp frameshift mutation that results in a reading frame shift. The primer sequences utilized in this study are detailed in Supplementary Table S1.

### 2.3. Histological Analysis and Germ Cell Counting

Male Nile tilapia from the experimental groups (six individuals per age stage for *cbx3a*^−/−^ mutants) and the control group (six WT individuals per age stage) were anesthetized using MS-222 (100 mg/L) prior to dissection for the collection of gonadal tissues. Body weight (BW) and gonad weight (GW) were measured to calculate the gonadosomatic index (GSI). Gonadal tissues were fixed in Bouin’s solution at room temperature for 12 h, followed by sequential dehydration in graded ethanol, clearing in xylene, and embedding in paraffin. Serial transverse sections (5 μm thickness) were prepared using a rotary microtome (Leica RM2235, Germany), mounted on slides, dried, and stained with hematoxylin and eosin (H&E). The stained sections were observed and imaged using an optical microscopy system (Olympus BX51, Japan).

For the quantitative analysis of spermatogenic cells, six H&E-stained sections (*n* = 6) from each group of WT males at 90 and 180 days after hatching (dah), as well as *cbx3a*^−/−^ males categorized into Type I (where partial germ cells exhibit defective meiosis accompanied by sporadic apoptosis) and Type II (characterized by complete meiotic arrest and universal apoptosis in all germ cells), were randomly selected. Six randomized fields per section were examined using a 40× objective lens, and the numbers of spermatogonia, spermatocytes, and spermatids were quantified for subsequent statistical analysis.

### 2.4. Fluorescent Immunohistochemistry

Male WT Nile tilapia at 5, 30, 90, and 180 dah (*n* = 5 per stage) were anesthetized, and their intact testes were dissected. The tissues were fixed in 4% paraformaldehyde (PFA) at 4 °C for 12 h, followed by gradient dehydration and paraffin embedding. Serial transverse sections of 5 μm thickness were prepared using a microtome. The sections were dewaxed and rehydrated, then incubated with rabbit anti-Cbx3a polyclonal antibody (1:500 dilution). Goat anti-rabbit Alexa Fluor 488- or 594-conjugated secondary antibodies (Thermo Fisher Scientific, USA) were diluted to 1:500 in blocking solution and incubated with the samples overnight at 4 °C to detect the primary antibodies. Nuclei were counterstained with 4′,6-diamidino-2-phenylindole (DAPI) (Vector Laboratories, USA). At least three independent biological replicates per genotype were analyzed, and fluorescence signals were captured using a confocal microscope (Olympus FV3000, Japan). The full-length open reading frame (ORF) of *cbx3a* was amplified using RT-PCR to generate a rabbit polyclonal antibody against Cbx3a. Sequence analysis shows that tilapia Cbx3a and its closely related paralog Cbx3b share approximately 67.8% overall amino acid identity, with ~90% identity within the highly conserved chromo domain. Therefore, we cannot formally exclude the possibility of cross-reactivity with Cbx3b. However, the absence of the expected ~21 kDa band in *cbx3a*^−/−^ testis lysates provides indirect evidence that the antibody predominantly recognises Cbx3a under our experimental conditions. This polyclonal antibody, custom-produced by Abiotech Co., Ltd. (Abiotech, China), was employed to validate the cellular localization of Cbx3a in tilapia gonads. The antigenic peptide sequence matched that found in the NCBI database (XP_005453878.1). Rabbit anti-γH2AX (1:200, Cell Signaling Technology, #9718) and Rabbit anti-Sycp3 (1:100, Santa Cruz Biotechnology, sc-74569) were used at 1:500 dilution. For quantification of γH2AX and Sycp3 immunofluorescence signals, six random microscopic fields per section were captured from each biological replicate (*n* = 6 per genotype). The percentage of positive cells was calculated by manually counting γH2AX- or Sycp3-positive cells relative to the total number of DAPI-stained cells within each field. To ensure objectivity, the counting was performed in a blinded manner with respect to the genotype. Data are presented as mean ± SD, and statistical significance was determined using one-way ANOVA followed by Tukey’s test.

### 2.5. Western Blot

Testes were dissected from 90 dah WT and *cbx3a*^−/−^ male fish (*n* = 6 per group) for total protein extraction. Protein concentrations were adjusted to a final concentration of 20 mg/mL. Proteins were separated using 12.5% SDS-PAGE gels and subsequently transferred to PVDF membranes via a semi-dry transfer system (LanJian FTB95, China) at a constant voltage of 25 V for 7.5 min. Membranes were blocked with 5% skimmed milk and incubated with the following antibodies: rabbit anti-Cbx3a polyclonal antibody (1:500 dilution), mouse anti-α-tubulin antibody (1:1000 dilution, internal control), and horseradish peroxidase (HRP)-conjugated goat anti-rabbit secondary antibody (Beyotime, China; 1:1000 dilution). Primary antibodies were diluted in blocking buffer (skimmed milk), while secondary antibodies were diluted in TBST. Signals were detected using a chemiluminescence imaging system (Tanon 6600, China).

### 2.6. Apoptosis Assay

Testes were dissected from 180 dah WT and *cbx3a*^−/−^ Type I and Type II male fish (*n* = 6 per group) for paraffin section preparation. Germ cell apoptosis was analyzed using the Terminal deoxynucleotidyl transferase dUTP nick end labeling (TUNEL) assay (Roche, Germany), in conjunction with fluorescent immunohistochemistry to detect the germ cell-specific marker, protein Vasa. The TUNEL assay was performed according to the manufacturer’s protocol, and fluorescent signals were captured using the aforementioned confocal microscopy system.

### 2.7. Sperm Morphology Staining

Semen was collected from 180 dah WT and *cbx3a*^−/−^ Type I and Type II male fish (*n* = 6). For sperm labeling, 20 μL of semen was mixed with 100 μL of PKH26 buffer and 1 μL of PKH26 fluorescent dye (Sigma, USA), followed by a 5-min incubation at room temperature in the dark. Sperm activation was achieved by adding 5 μL of sterile water. The resulting fluorescent signals were captured using a confocal microscopy system (Olympus FV3000, Japan).

### 2.8. Sperm Motility Analysis

Semen was collected from 180 dah WT and *cbx3a*^−/−^ Type I and Type II male fish (*n* = 6). Sperm motility parameters, including movement trajectories, beat-cross frequency (BCF), curvilinear velocity (VCL), and straight-line velocity (VSL), were analyzed using a computer-assisted sperm analysis (CASA) system (ZKPACS-E, Leica, Germany). Raw semen samples were diluted 20-fold with deionized water, loaded into a sperm counting chamber, and subjected to microscopic observation, imaging, and analysis. Each sample was analyzed in triplicate. To minimize the impact of sperm inactivation on data accuracy, image acquisition was completed within 5 s, and three randomly selected fields per sample were recorded for statistical analysis.

### 2.9. Transcriptome Sequencing and Analysis

Testes were harvested separately from WT and *cbx3a* homozygous mutant specimens at 60 dah. Total RNA was extracted from each pooled sample (each pool containing testes from at least 6 fish per genotype, with three independent pools per genotype). Six libraries were constructed (three from WT pools and three from mutant pools) and sequenced using the Illumina NGS system (Personal Biotech, Nanjing, China). All clean reads were mapped to the tilapia reference genome (O_niloticus_UMD_NMBU, GCA_001858045.3) using HISAT2 (v2.1.0). Gene expression levels were quantified as fragments per kilobase of exon per million fragments mapped (FPKM) using StringTie (v2.1.4). Differentially expressed genes were identified using DESeq2 (v1.30.0) with an absolute |log_2_(fold change)| > 1 and Benjamini–Hochberg-adjusted *p* < 0.05 as the significance threshold. All the raw data were deposited in the NCBI Short Read Archive (accession number: PRJNA906360).

### 2.10. Statistical Analysis

Data were analyzed using GraphPad Prism 8 software and expressed as the mean ± standard deviation (SD). For comparisons involving more than two groups, one-way ANOVA followed by Tukey’s test was used, and significance (*p* < 0.05) is indicated by distinct letters. For comparisons between two groups (WT versus *cbx3a*^−/−^), unpaired two-tailed Student’s *t*-test was used, and significance is indicated as * *p* < 0.05, ** *p* < 0.01, and *** *p* < 0.001.

## 3. Results

### 3.1. Cell Expression Patterns of Cbx3a in the Testis of Nile Tilapia

**Figure 1:**
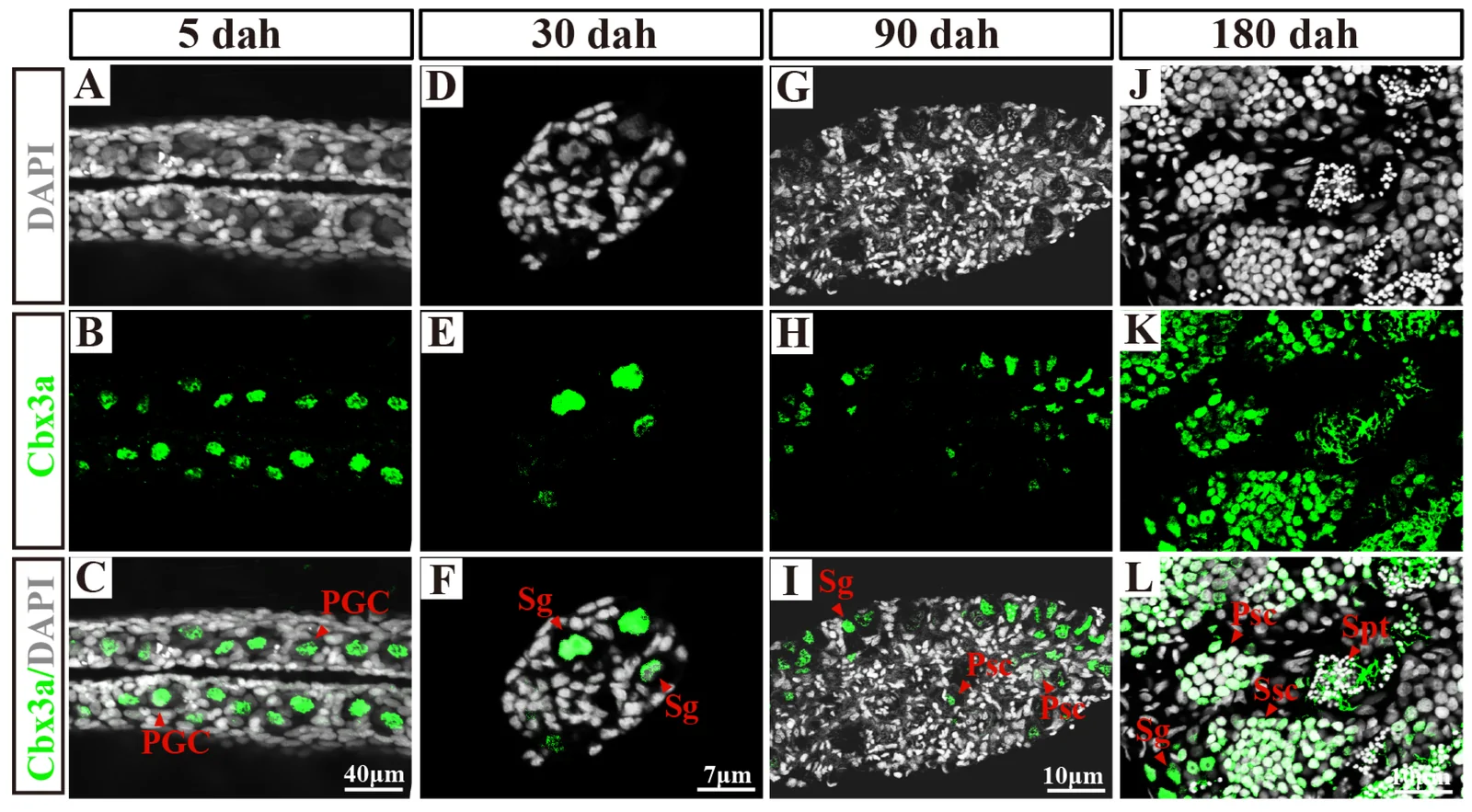
Cellular expression and localization of Cbx3a in Nile tilapia testes during different developmental stages. (**A**–**C**) Cbx3a was specifically localized in the nuclei of germ cells within undifferentiated testes. (**D**–**F**) Additionally, Cbx3a was detected in the nuclei of spermatogonia in these undifferentiated testes (Sg). (**G**–**I**) During the early stages of meiosis, Cbx3a expression was observed in both spermatogonia and primary spermatocytes (Psc). (**J**–**L**) In sexually mature testes, Cbx3a was expressed in the nuclei of all types of spermatogenic cells. Gray fluorescence indicates DAPI signals, while green fluorescence represents Cbx3a signals. Sg, spermatogonia; Psc, primary spermatocytes; Ssc, secondary spermatocytes; Spt, spermatids; dah, days after hatching.

At the early developmental stage (5 dah), distinct Cbx3a immunoreactive signals were detected in cells displaying PGC morphology and positional characteristics within the nascent gonadal primordium (Figure 1A–C), suggesting its potential role in germline specification. During spermatogenic progression (30–180 dah), Cbx3a exhibited stage-specific nuclear localization across the spermatogenic stages: in spermatogonia, robust expression was observed in both undifferentiated and differentiating spermatogonia (Figure 1D–F). In the meiotic phase, sustained signal intensity was noted in primary and secondary spermatocytes (Figure 1G–I), indicating its functional relevance during meiosis. In haploid differentiation, persistent nuclear staining was observed in spermatids (Figure 1J–L), suggesting roles in post-meiotic chromatin remodeling.

### 3.2. Generation of Cbx3a Mutants

**Figure 2:**
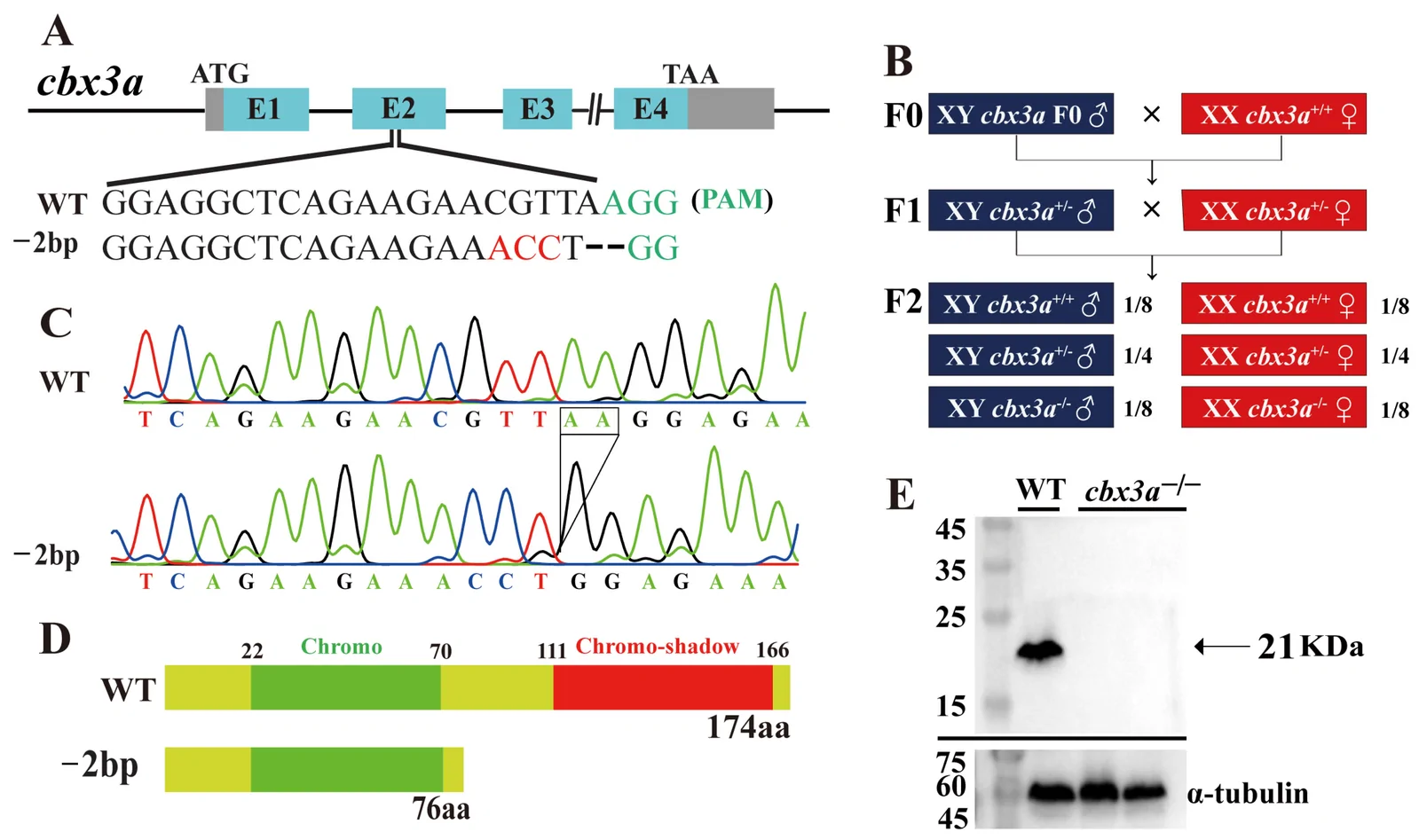
Target site design, generation and validation of *cbx3a* homozygous knockout mutants. (**A**) The gene structure of *cbx3a* and details regarding the mutation target sites are presented. The knockout target sites are situated within the second exon, with the PAM (Protospacer Adjacent Motif) region highlighted in green. (**B**) The methodology employed for generating *cbx3a* homozygous mutants is outlined. (**C**) A sequence chromatogram is provided to illustrate the target site in *cbx3a* homozygous mutants. (**D**) A schematic diagram is presented, depicting the domains of the Cbx3a protein; the Chromo-domain is indicated in green, while the Chromo-shadow domain is represented in red. (**E**) Validation of *cbx3a* homozygous mutants was conducted through Western blot analysis, utilizing α-tubulin as the internal control. The original Western blot images can be found in the Supplementary Materials (Figure S1).

A single-guide RNA (sgRNA) targeting the second exon of *cbx3a* (sequence: 5′-GGAGGCTCAGAAGAAGAACGTTAAGG-3′) was designed (Figure 2A). The sgRNA and Cas9 mRNA were co-injected into fertilized eggs at the one-cell stage via microinjection. Genomic DNA extracted from 30 injected embryos at 24 h post-fertilization (hpf) was subjected to PCR amplification, followed by HMA and Sanger sequencing to identify mosaic F0 mutants (Figure 2B). Sexually mature F0 XY founders harboring germline mutations were outcrossed with WT XX females to produce F1 heterozygous offspring. HMA screening of F1 progeny revealed diverse mutation patterns, from which individuals carrying a 2-bp deletion in *cbx3a* were selected for subsequent analysis. Subcloning and sequencing confirmed that the deletion caused a frameshift mutation, resulting in premature termination of translation and the loss of the chromo-shadow domain critical for HP1γ function (Figure 2C,D). Homozygous *cbx3a* mutants (*cbx3a*^−/−^) were established through incrossing of the F1 mutants. Western blot analysis of gonadal lysates from WT and *cbx3a*^−/−^ individuals revealed a specific 21-kDa band in WT samples, which was absent in mutants (Figure 2E), confirming the successful ablation of Cbx3a protein in the mutant line.

### 3.3. Significant Reduction in Spermatogenic Cells in Cbx3a Mutants

**Figure 3:**
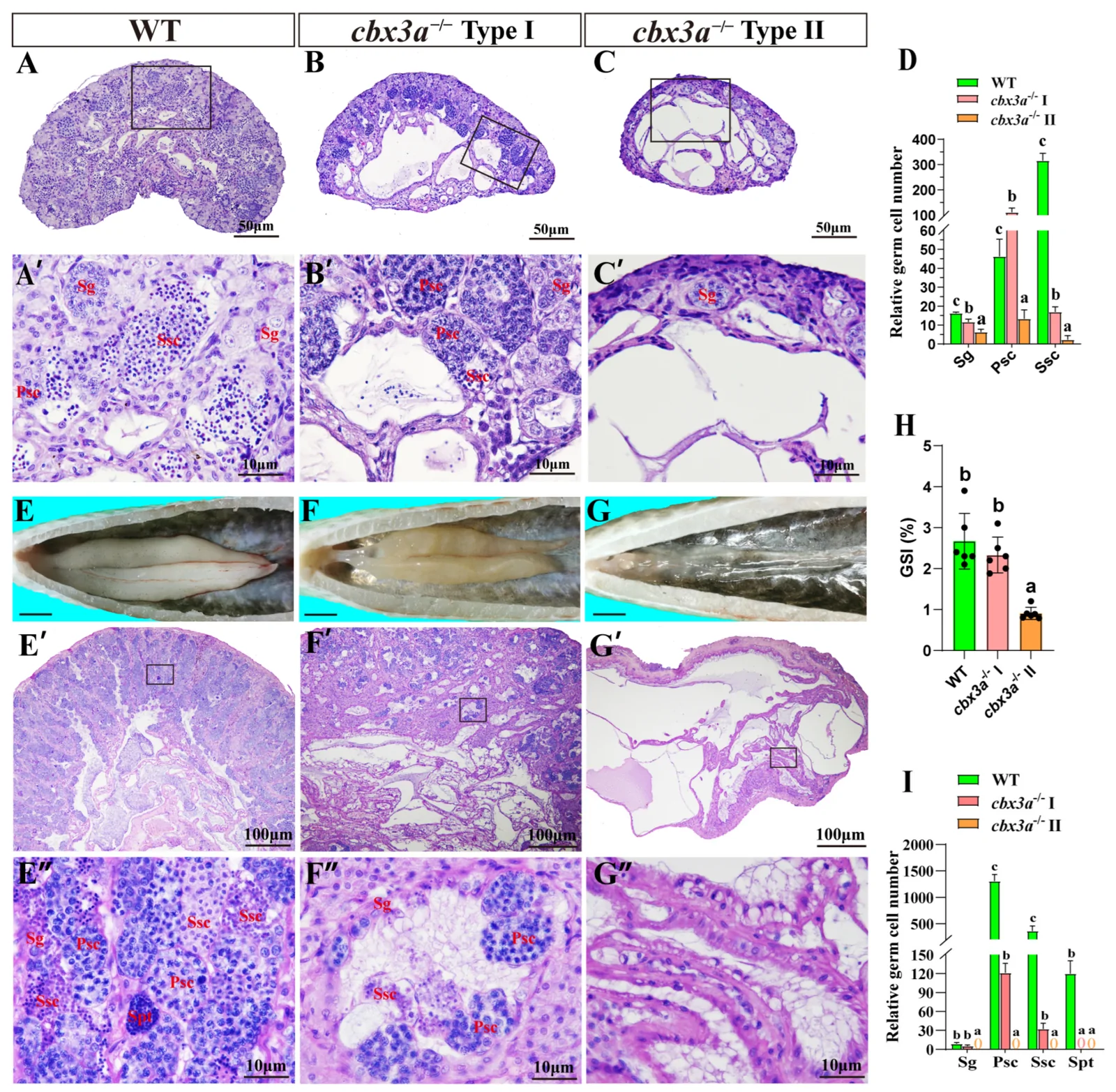
Morphological and histological phenotypes of testes in *cbx3a^−/−^* male tilapia. (**A**–**C**) Testicular phenotypes observed in WT and *cbx3a^−/−^* males at 90 dah. The mutants displayed two distinct phenotypes: one characterized by partial germ cell loss (**B**,**B′**), and another exhibiting a majority of germ cell depletion (**C**,**C′**). (**D**,**I**) Germ cell counts. For each cell type, different letters above the bars indicate significant differences (*p* < 0.05) among the three genotypes as determined by one-way ANOVA followed by Tukey’s test. Comparisons are made only within the same cell type, not across different cell types. (**E**–**G**) Testicular phenotypes in WT and *cbx3a^−/−^* males at 180 dah. (**H**) Gonadosomatic index (GSI). Data are expressed as mean ± standard deviation (SD) from triplicate measurements. Statistical significance is indicated by different letters above the error bars (*p* < 0.05). Magnified views of the boxed regions in panels (**A**–**C**) and (**E**–**G**) are provided in (**A′**–**C′**) and (**E′**–**G′**), respectively. (**E″**–**G″**) are higher-magnification views of the boxed areas in (**E′**–**G′**), respectively. Abbreviations: Sg, spermatogonia; Psc, primary spermatocytes; Ssc, secondary spermatocytes; Spt, spermatids.

Analysis of testes from WT and *cbx3a*^−/−^ mutants (15 individuals per group) at 90 dah via H&E staining revealed that WT testes contain various spermatogenic cell lineages, including spermatogonia, primary spermatocytes, and secondary spermatocytes (Figure 3A,A′). In contrast, *cbx3a*^−/−^ mutants displayed two distinct phenotypic subtypes: approximately 8 out of 15 mutants (Type I) retained spermatogonia, primary spermatocytes, and secondary spermatocytes but exhibited partial cystic degeneration in the dorsal seminiferous tubules, characterized by variable reductions in germ cell numbers (Figure 3B,B′). The remaining 7 out of 15 mutants (Type II) showed no meiotic germ cells and displayed severe germ cell depletion, with only residual spermatogonia and no detectable spermatocytes (Figure 3C,C′). Statistical analysis indicated that, compared to WT, Type I mutants exhibited significantly reduced numbers of spermatogonia and secondary spermatocytes (*p* < 0.01), while primary spermatocytes were significantly increased (*p* < 0.05) (Figure 3D).

To investigate the persistence of mutant phenotypes, 180 dah WT and *cbx3a*^−/−^ individuals (six per group) were subjected to morphological and histological analyses. The results revealed sustained bifurcated phenotypes: Type I mutant testes exhibited hydration, translucency, and a pale pink hue at the gonad-anus junction (Figure 3E). In contrast, Type II mutants displayed significant testicular atrophy, characterized by a markedly reduced gonadosomatic index (GSI, *p* < 0.001) and abnormal morphology (Figure 3F). Histological examination confirmed that WT testes retained intact seminiferous tubules containing complete spermatogenic cell populations, including spermatogonia, all stages of spermatocytes, spermatozoa, and mature sperm (Figure 3E′,E″). Conversely, Type I mutants exhibited cystic degeneration in numerous tubules with diminished germ cell populations (Figure 3F′,F″), while Type II mutants displayed nearly complete germ cell loss, with only minimal or no germ cells remaining (Figure 3G′,G″).

### 3.4. Gene Expression Patterns in the Testes of Cbx3a Mutants

**Figure 4:**
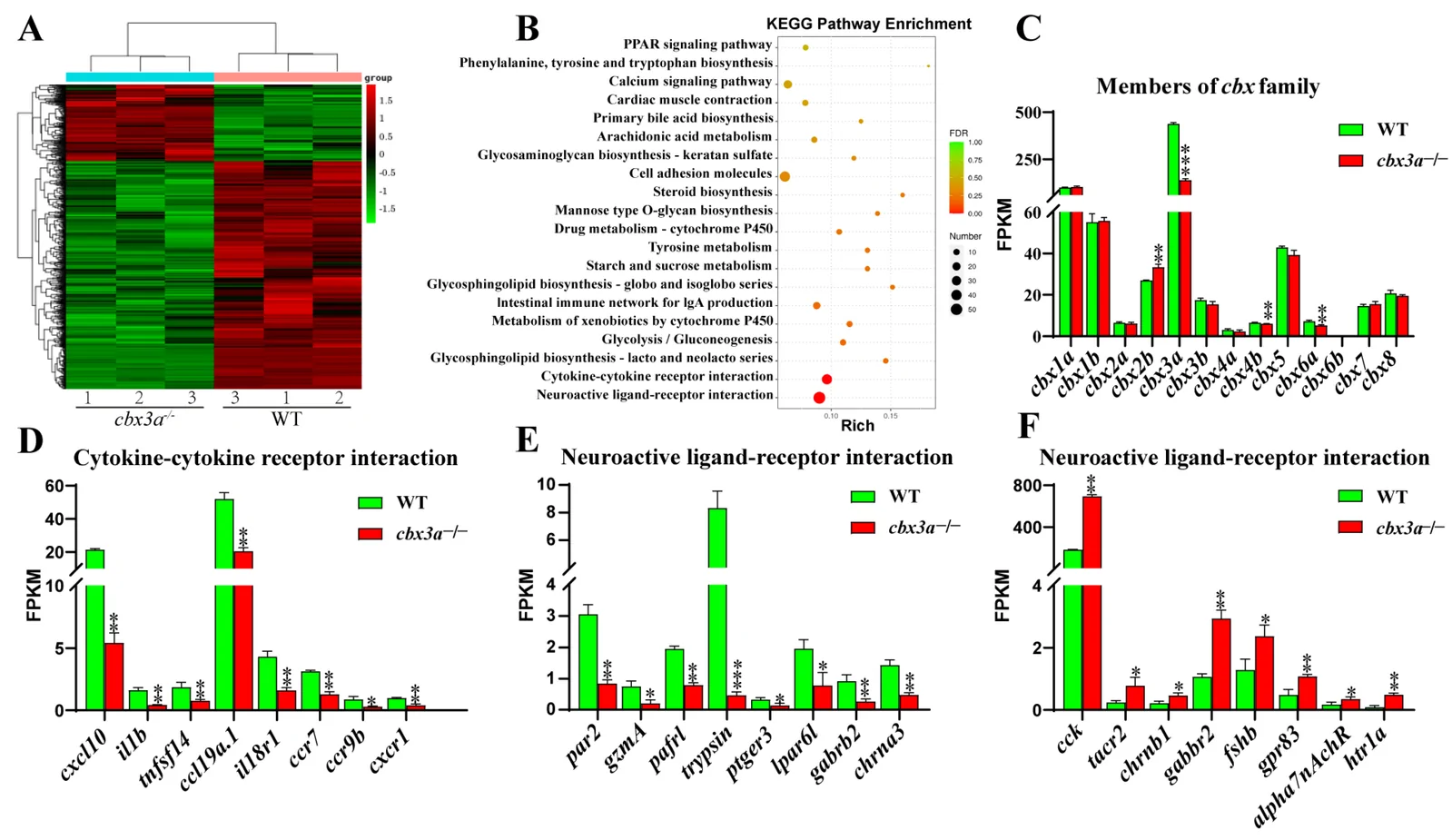
Comparative transcriptomic analysis of dysregulated genes and signaling pathways in *cbx3a^−/−^* tilapia. (**A**) A heatmap illustrating the differences in gene expression between testes from wild-type (WT) and *cbx3a*^−/−^ fish at 90 dah. (**B**) A scatter plot depicting the enriched KEGG pathways for differentially expressed genes (DEGs), where the sizes and colors of the dots represent the number of genes and the significance of the differences, respectively. (**C**) The effects of the *cbx3a*^−/−^ mutation on the expression of genes within the Cbx family. (**D**–**F**) Expression profiles of genes in representative significantly different signaling pathways. The data are presented as the mean ± SD of triplicates. Differences between groups were statistically analyzed using a two-tailed unpaired Student’s *t*-test, with significant differences denoted by * (*p* < 0.05), ** (*p* < 0.01) and *** (*p* < 0.001). The complete list of DEGs, including 333 upregulated and 992 downregulated genes, is provided in Supplementary Table S2.

Cluster analysis of differentially expressed genes (DEGs) between WT and *cbx3a*^−/−^ XY Nile tilapia testes revealed consistent transcriptional differences, with significant divergences in gene expression patterns between mutants and WT individuals (Figure 4A). Differentially expressed genes were classified based on the following criteria: |log_2_(*cbx3a*^−/−^ FPKM/WT FPKM)| > 1 and FDR (Benjamini–Hochberg-adjusted *p*) < 0.05. Under these criteria, a total of 333 genes were identified as significantly upregulated and 992 genes as significantly downregulated in *cbx3a*^−/−^ testes compared to WT, as detailed in Supplementary Table S2. KEGG pathway enrichment analysis of DEGs between WT and *cbx3a*^−/−^ XY testes, focusing on the top 20 pathways ranked by the lowest *p*-value, identified significantly impacted pathways, including neuroactive ligand–receptor interaction, cytokine-cytokine receptor interaction, glycosphingolipid biosynthesis (lacto and neolacto series), and glycolysis/gluconeogenesis (Figure 4B). Expression analysis of cbx family members in *cbx3a*^−/−^ versus WT testes revealed no significant changes in *cbx1a*, *cbx1b*, *cbx2a*, *cbx3b*, *cbx4a*, *cbx5*, *cbx6b*, *cbx7*, or *cbx8*. In contrast, *cbx2b* was upregulated, while cbx4b and cbx6a were downregulated (Figure 4C). Genes involved in the cytokine-cytokine receptor interaction pathway were significantly downregulated in *cbx3a*^−/−^ testes (Figure 4D). Within the neuroactive ligand–receptor interaction pathway, key genes such as *par2*, *gamA*, *pafrl*, *trypsin*, *ptger3*, *lpar6l*, *gabrb2*, and *chrna3* were significantly downregulated (Figure 4E). Notably, Trypsin, a critical multifunctional factor in spermatogenesis and closely linked to meiosis in teleosts [29], exhibited highly significant downregulation. Conversely, other genes in the same pathway, including *cck*, *tacr2*, *chrnb1*, *gabbr2*, *fshb*, *gpr83*, *alpha7nAchR*, and *htr1a*, were significantly upregulated in mutant testes (Figure 4F).

### 3.5. Increased Apoptosis of Germ Cells in Cbx3a^−/−^ Testis

**Figure 5:**
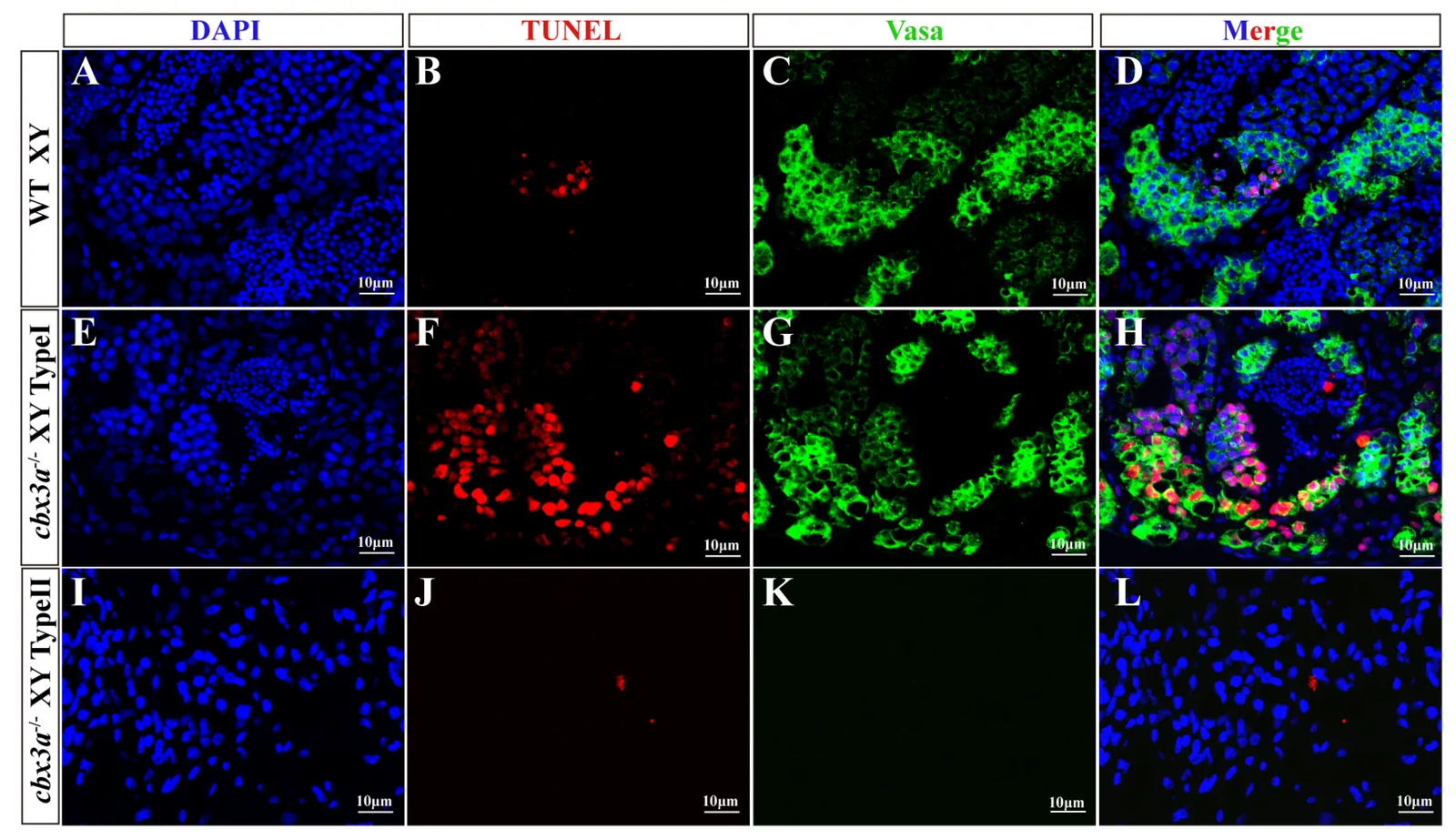
Testicular cell apoptosis detection in *cbx3a^−/−^* tilapia. (**A**–**D**) WT testis: (**A**) DAPI nuclear staining; (**B**) TUNEL-positive apoptotic signals; (**C**) Vasa-positive germ cells; (**D**) merged image. (**E**–**H**) *cbx3a*^−/−^ Type I testis: (**E**) DAPI; (**F**) TUNEL; (**G**) Vasa; (**H**) merge. (**I**–**L**) *cbx3a*^−/−^ Type II testis: (**I**) DAPI; (**J**) TUNEL; (**K**) Vasa; (**L**) merge. Blue, red, and green fluorescence indicate DAPI, TUNEL, and Vasa signals, respectively.

The *cbx3a*^−/−^ fish exhibited a significant absence of germ cells compared to the WT fish, prompting speculation regarding the occurrence of abnormal apoptosis of germ cells in the testis. To investigate this hypothesis, we assessed germ cell apoptosis in 180 dah *cbx3a*^−/−^ fish using TdT-mediated dUTP nick-end labeling (TUNEL) combined with Vasa fluorescence immunohistochemistry, a known germ cell marker. A substantial number of apoptotic germ cells were observed in *cbx3a*^−/−^ Type I individuals compared to the WT group (Figure 5A–H). In *cbx3a*^−/−^ Type II individuals, both TUNEL and Vasa positive signals were absent, which aligns with the nearly complete lack of germ cells observed in H&E staining (Figure 5I–L). These findings suggest that mutations in *cbx3a* lead to extensive apoptosis of spermatogenic cells, resulting in a significant reduction in germ cells in the testes of *cbx3a*^−/−^ individuals.

### 3.6. Abnormal Morphology and Decreased Motility of Sperm from Cbx3a Mutants

**Figure 6:**
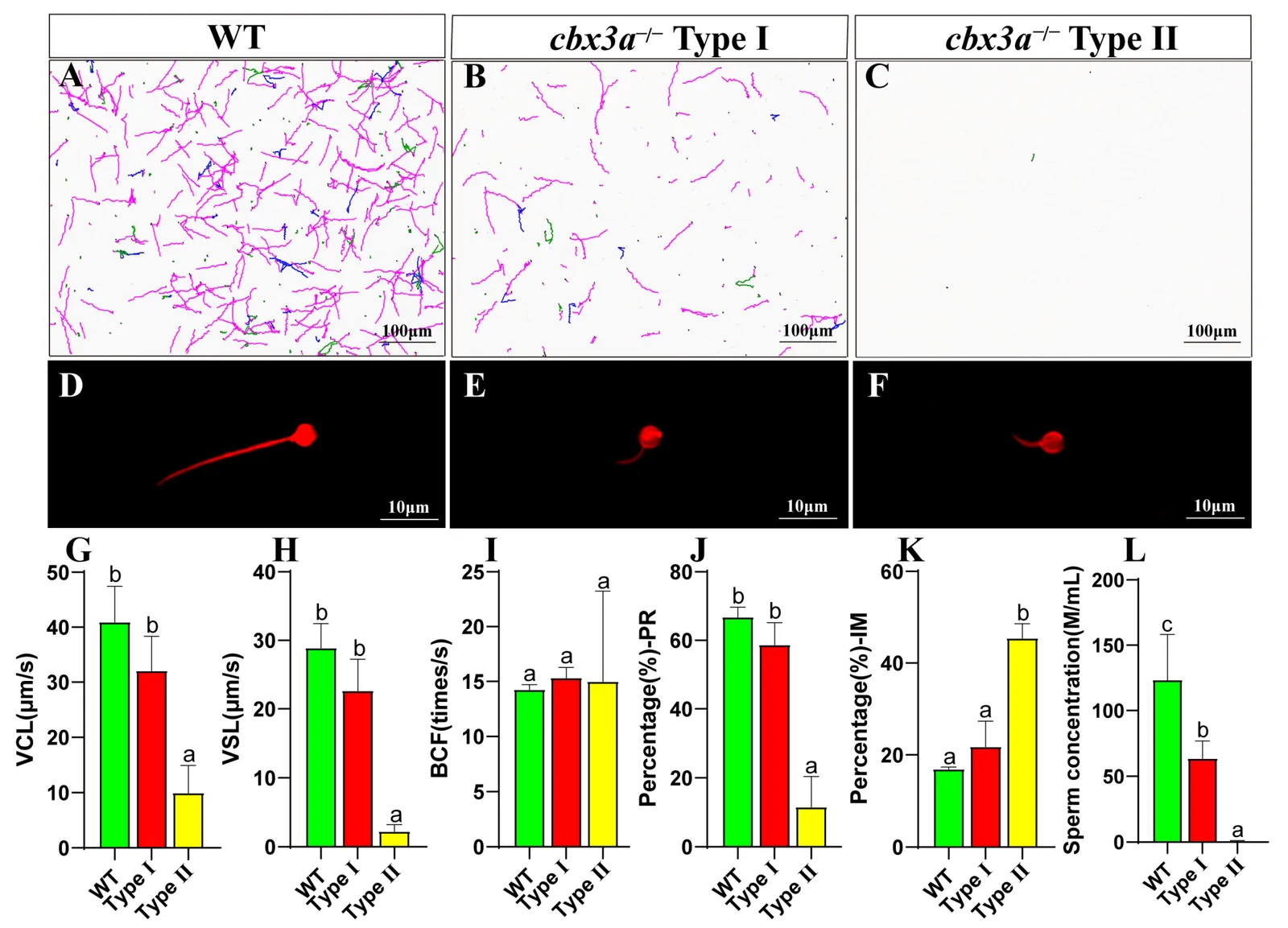
Analysis of sperm motility and morphology in *cbx3a*^−/−^ tilapia. (**A**–**C**) The trajectory curves of WT and *cbx3a*^−/−^ sperm from fish at 180 dah are presented. (**D**–**F**) The morphology of sperm from WT and *cbx3a*^−/−^ fish is depicted, with red fluorescence indicating the PKH26 signal. (**G**) Curvilinear velocity is measured. (**H**) Straight-line velocity is assessed. (**I**) The beat frequency of sperm flagella is analyzed. (**J**) The proportion of progressive sperm is quantified. (**K**) The count of immotile sperm is recorded. (**L**) Sperm concentration is evaluated. The purplish-red curve illustrates the trajectory of normal sperm, while the blue curve represents the trajectory of relatively normal sperm. In contrast, the green and black curves denote the trajectory of sperm exhibiting dysmotility. Results are expressed as the mean ± standard deviation. Statistical differences at *p* < 0.05, as determined by One-way ANOVA followed by Tukey’s test, are indicated by different letters above the error bars.

To assess the impact of the *cbx3a* homozygous mutation on sperm quality, we analyzed sperm motility parameters in WT and *cbx3a*^−/−^ mutants using a Computer-Assisted Sperm Analysis (CASA) system. The results revealed that in *cbx3a*^−/−^ Type I individuals, key motility parameters—including VCL, VSL, BCF, PR, and IM—showed no statistically significant differences compared to WT. However, sperm concentration was significantly reduced in Type I mutants (Figure 6A–C,G–L). Real-time motion trajectory observation, conducted under the same dilution factor, indicated that Type I mutants had significantly fewer viable sperm compared to WT (Figure 6A,B). Notably, in *cbx3a*^−/−^ Type II individuals, critical motility metrics (VCL, VSL, PR, and sperm concentration) were markedly lower than those observed in WT (Figure 6G,H,J,L), with only a few sperm detectable in the seminal fluid (Figure 6A–C).

To further investigate structural abnormalities, mature sperm were stained with the viable cell dye PKH26 for morphological observation. Compared to WT sperm, a majority of sperm from Type I mutants exhibited significant morphological abnormalities, including shortened tails (Figure 6D,E). Additionally, the rare residual sperm from Type II mutants also displayed abnormal short-tail morphology (Figure 6D–F). These findings suggest that *cbx3a* deficiency impairs male fertility through two primary mechanisms: a marked reduction in spermatogenic output and the presence of morphological abnormalities in sperm tails. The synergistic effects of these mechanisms ultimately lead to decreased sperm motility.

### 3.7. Impaired Meiotic Progression in Cbx3a Mutants

**Figure 7:**
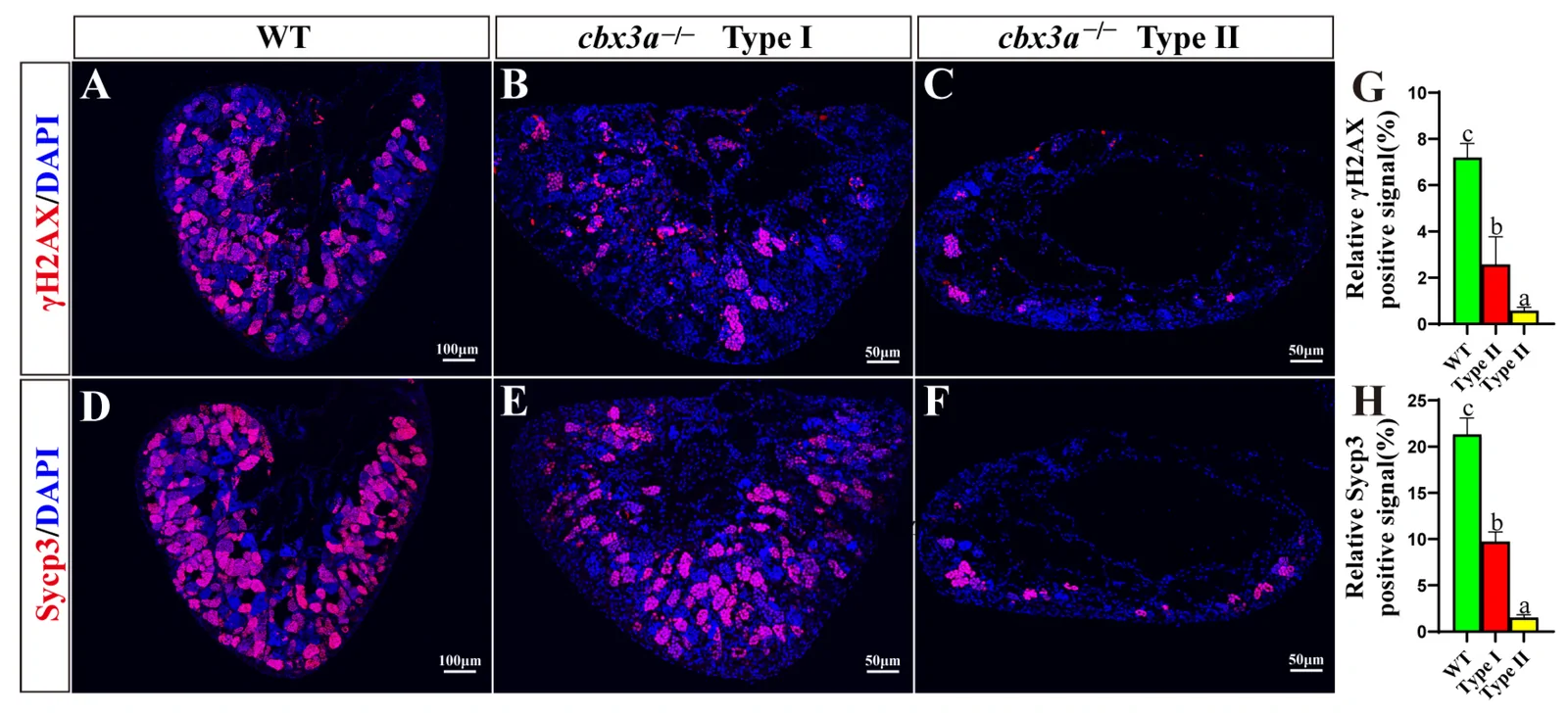
Expression patterns of meiotic marker genes in the testis of *cbx3a*^−/−^ tilapia. (**A**–**F**) Detection of γH2AX and Sycp3 expression in the testis was conducted using fluorescent immunohistochemistry. Red fluorescence indicates positive signals for γH2AX (**A**–**C**) and Sycp3 (**D**–**F**), while blue fluorescence corresponds to DAPI staining. (**G**,**H**) Statistical analysis of the immunofluorescence positive signals. Results are expressed as the mean ± standard deviation. Different letters above the error bar indicate statistical differences at *p* < 0.05, as determined by One-way ANOVA followed by Tukey’s test. The Y-axis in (**G**,**H**) represents the percentage of γH2AX- or Sycp3 positive cells relative to total DAPI positive cells, which was obtained by manual counting in a blinded manner from six random fields per section, with *n* = 6 biological replicates per genotype.

Histological analysis of the gonads revealed that the homozygous mutation of *cbx3a* resulted in a significant loss of germ cells and impaired spermatogenesis. To assess whether meiotic progression was affected, we examined the expression of two key meiotic markers, γH2AX (a marker for DNA double-strand breaks during early meiotic prophase) and Sycp3 (a component of the synaptonemal complex). Fluorescence immunohistochemistry and statistical analysis revealed that the percentages of γH2AX- and Sycp3-positive germ cells were significantly reduced in the testes of both Type I and Type II *cbx3a*^−/−^ individuals compared to WT (Figure 7A–F). Notably, this reduction is consistent with a marked decrease in the number of germ cells that initiate or enter meiosis, rather than a specific arrest at a particular meiotic stage (which would have been expected to cause accumulation of these markers). These results indicate that Cbx3a deficiency leads to impaired meiotic initiation or early meiotic progression, likely as a consequence of profound germ cell depletion.

## 4. Discussion

This study systematically elucidates the critical regulatory role of Cbx3a (a teleost-specific paralog of HP1γ) in the spermatogenesis of Nile tilapia through a comprehensive approach that includes cellular, histological, transcriptomic, and functional analyses. Utilizing CRISPR/Cas9-mediated gene knockout, histological examination, transcriptome sequencing, and functional validation, we demonstrate that the deficiency of *cbx3a* results in various impairments in spermatogenesis, such as germ cell apoptosis, meiotic arrest, and structural defects in sperm. These findings not only enhance our understanding of the functional evolution of HP1γ in vertebrates but also underscore its regulatory role in balancing conservation and divergence to maintain germ cell integrity.

### 4.1. Evolutionary Conservation and Divergence of HP1γ Function

In mammals, HP1γ (Cbx3) is well established as a key factor in heterochromatin formation, gene silencing, and meiotic chromosome dynamics [21,22,30]. This study demonstrates that Cbx3a in Nile tilapia, the teleost ortholog of mammalian Cbx3, also plays an indispensable role in germ cell survival and meiosis, reflecting a high degree of evolutionary conservation. For instance, the severe germ cell depletion and meiotic arrest observed in *cbx3a* homozygous mutant tilapia closely resemble the phenotypes reported in *Cbx3*-knockout mice [31].

However, due to the third round of whole-genome duplication (3R WGD), teleosts have retained two paralogous genes, *cbx3a* and *cbx3b*, providing an evolutionary basis for functional divergence. During spermatogenesis, *cbx3a* is predominantly expressed, while *cbx3b* exhibits very low expression levels, suggesting subfunctionalization or neofunctionalization [26]. It should be noted that while our antibody specifically detected a single band of the expected size in WT testes that was absent in *cbx3a*^−/−^ mutants, we cannot formally rule out cross-reactivity with the closely related Cbx3b paralog (sharing ~90% identity within the chromo domain). Future studies using peptide competition assays or paralog-specific knockdown/overexpression experiments would further strengthen the specificity conclusion. This divergence may reflect adaptive strategies to optimize reproductive success in aquatic environments. Notably, *cbx3a* homozygous mutants display varying degrees of testicular defects, which we classify into two subtypes: Type I and Type II. We initially speculated that these differences might arise from variations in developmental timing; however, the phenotypic divergence persists even after sexual maturation. Therefore, we propose that the heterogeneity of spermatogenic defects caused by the *cbx3a* mutation may originate from inter-individual genetic or epigenetic variation, and the underlying mechanisms warrant systematic investigation in future studies.

### 4.2. Mechanisms Underlying Germ Cell Apoptosis and Disrupted Meiosis in Homozygous Mutants

Transcriptomic analysis revealed a significant dysregulation of pathways closely related to germ cell homeostasis in *cbx3a* mutants, particularly the cytokine-cytokine receptor interaction pathway and the neuroactive ligand–receptor interaction pathway. Notably, the downregulation of trypsin is especially significant; this protease plays a critical role in meiotic progression in teleosts [29,32,33,34], and its decreased expression likely contributes to the failure of synaptonemal complex formation and subsequent meiotic arrest. Additionally, the marked reductions in γH2AX (a marker of DNA double-strand breaks in primary spermatocytes during the leptotene and zygotene stages of meiosis) and Sycp3 (which is involved in synaptonemal complex assembly and is primarily expressed in early primary spermatocytes) [35,36,37] further confirm the critical role of Cbx3a in maintaining meiotic fidelity, likely by ensuring the proper initiation and progression of meiosis in sufficient numbers of germ cells.

The pronounced germ cell apoptosis observed in Type I mutants, alongside the near-complete germ cell loss in Type II mutants, is closely associated with disrupted cytokine signaling [38]. The downregulation of *il6st* and *csf3r* [39,40] in this study further supports this association. Cytokines are known to mediate the crosstalk between germ cells and supporting cells [41,42], and their deficiency may destabilize the spermatogonial stem cell niche, thereby inducing apoptosis [43,44]. Notably, the number of primary spermatocytes was significantly increased in Type I mutants. This seemingly paradoxical phenomenon suggests the existence of a compensatory mechanism attempting to rescue meiotic progression, albeit unsuccessfully; alternatively, it may indicate that most primary spermatocytes fail to advance properly to the secondary spermatocyte stage. This phenomenon warrants further investigation using lineage tracing or single-cell sequencing.

### 4.3. Structural and Functional Defects in Spermatozoa of cbx3a Mutants

The impact of Cbx3a deficiency on spermatogenesis exhibits a dual nature characterized by both quantity and quality. On one hand, there is a significant reduction in sperm production; on the other hand, notable morphological abnormalities in the sperm tail are observed. The short-tailed sperm identified through PKH26 staining likely results from defective post-meiotic chromatin remodeling [45], a process that relies on HP1γ-mediated heterochromatin organization [46]. Additionally, the substantial decrease in sperm motility parameters (VCL, VSL, PR) in Type II mutants underscores the essential role of Cbx3a in sustaining cytoskeletal integrity and energy metabolism [47,48]. Furthermore, the dysregulation of the glycolysis/gluconeogenesis pathway in the transcriptome provides additional support for this perspective.

## 5. Conclusions

In summary, Cbx3a in Nile tilapia is a crucial regulator of male gametogenesis, coordinating germ cell survival, meiotic progression, and sperm morphogenesis through a complex epigenetic and signaling network. Its functional conservation with mammalian HP1γ highlights the ancient evolutionary origin of heterochromatin-mediated reproductive regulation. Concurrently, the lineage-specific adaptation of Cbx3a in teleosts presents new avenues for fundamental research and germplasm innovation in aquaculture. In this study, we successfully generated a *cbx3a* homozygous mutant in tilapia using CRISPR/Cas9 technology. Our findings provide a theoretical foundation for enhancing sperm quality or controlling fertility in farmed fish by modulating *cbx3a*, particularly in species where monosex culture is advantageous. Furthermore, the conserved function of HP1γ in vertebrate meiosis suggests that insights gained from tilapia could inform research in other economically significant fish species and even in mammalian models of infertility. It should be noted that our conclusions regarding reproductive capacity are based on gametic parameters (sperm concentration, motility, and morphology). Direct mating trials are warranted in future studies to quantitatively assess fertilisation success and to validate the potential for sterile line development. Although this study has identified key pathways affected by Cbx3a deficiency, the specific molecular interactions between Cbx3a and downstream targets, such as trypsin and cytokine receptors, require further validation. Future studies should utilize chromatin immunoprecipitation sequencing (ChIP-seq) to systematically map the genome-wide binding profile of Cbx3a and identify its direct regulatory targets. Meanwhile, investigating the compensatory roles of Cbx3b and other chromatin modifiers, such as DNA methyltransferases, will help elucidate functional redundancy and synergistic mechanisms in teleost spermatogenesis.

## Data Availability

The raw sequencing data of transcriptome generated in this study are openly available in the NCBI Short Read Archive (accession number: PRJNA906360).

## Supplementary Materials

The following supporting information can be downloaded at: https://www.mdpi.com/article/10.3390/biom16081120/s1; Figure S1 Original Western blot images; Table S1: Primers used in present study; Table S2: C3a-KO_vs_C3a-WT DESeq Down and UP.

## Abbreviations

The following abbreviations are used in this manuscript:

HP1	Heterochromatin Protein 1
CRISPR/Cas9	Clustered regularly interspaced short palindromic repeats -associated protein 9
PCH	pericentric heterochromatin
3R WGD	the third round of whole-genome duplication
gRNA	guide RNA
dpf	days post-fertilization
HMA	heteroduplex mobility assay
WT	wild-type
BW	Body weight
GW	gonad weight
GSI	gonadosomatic index
H&E	hematoxylin and eosin
dah	days after hatching
PFA	paraformaldehyde
DAPI	4′,6-diamidino-2-phenylindole
ORF	open reading frame
SDS-PAGE	Sodium Dodecyl Sulfate—Polyacrylamide Gel Electrophoresis
PVDF	Polyvinylidene Difluoride
HRP	horseradish peroxidase
TUNEL	Terminal deoxynucleotidyl transferase dUTP nick end labeling
BCF	beat-cross frequency
sgRNA	single-guide RNA
hpf	hours post-fertilization
SD	standard deviation
Sg	spermatogonia
Psc	primary spermatocytes
Ssc	secondary spermatocytes
Spt	spermatids
DEGs	differentially expressed genes
ChIP-seq	chromatin immunoprecipitation sequencing
