# Supplementary material for: Cbx3a/HP1γ Deficiency Disrupts Meiotic Progression and Triggers Germ Cell Apoptosis in Nile Tilapia
Source: Biomolecules. 2026 Jul 31;16(8):1120. doi: 10.3390/biom16081120 (PMC13510707; doi:10.3390/biom16081120)
Supplement: Supplementary file 1 [file biomolecules-16-01120-s001.zip › Supplementary Table S1.pdf]

Supplementary Table S1 **Primers used in present study**

Primer	Sequence（5'-3'）	Purpose
***cbx3a*-gRNA-F**	TAATACGACTCACTATAGGAGGCTCAGAAGAACGTTAGTTTTAGAGCTAGAAATAGC	CRISPR/Cas9
**gRNA-R**	AGCACCGACTCGGTGCCAC	
***cbx3a*-PAGE-F**	AGAGGAGAACCTGGATTGCCC	Mutants screening by HMA
***cbx3a*-PAGE-R**	GCCTTCCTCTTAACAGGCGC	
***cbx3a*-T-F**	GCTGGAAGTGCTTCCCTGGT	Mutants screening by Sanger sequencing
***cbx3a*-T-R**	CCCGTGTTAAGCTTTGCTGTCC	
***amh*-F5**	ATGGCTCCGAGACCTTGACTG	Sex genotyping
***amh*-R3**	CAGAAATGTAGACGCCCAGGTAT	
